# Knockdown of menin affects pre-mRNA processing and promoter fidelity at the interferon-gamma inducible *IRF1 *gene

**DOI:** 10.1186/1756-8935-5-2

**Published:** 2012-01-12

**Authors:** Lauren B Auriemma, Shaili Shah, Lara M Linden, Melissa A Henriksen

**Affiliations:** 1 Department of Biology, The University of Virginia, 485 McCormick Road, Charlottesville, VA 22903, USA; 2 Hoffman-La Roche, 340 Kingsland Road, Building 102, Room C519, Nutley, NJ 07110, USA

**Keywords:** HDACs, histone, JAK-STAT, lysine methylation, transcription

## Abstract

**Background:**

The tumor suppressor menin (*MEN1*) is mutated in the inherited disease multiple endocrine neoplasia type I, and has several documented cellular roles, including the activation and repression of transcription effected by several transcription factors. As an activator, MEN1 is a component of the Set1-like mixed lineage leukemia (MLL) MLL1/MLL2 methyltransferase complex that methylates histone H3 lysine 4 (H3K4). MEN1 is localized to the signal transducer and activator of transcription 1 (STAT1)-dependent gene, interferon regulatory factor 1 (*IRF1)*, and is further recruited when *IRF1 *transcription is triggered by interferon-γ signaling.

**Results:**

RNAi-mediated knockdown of MEN1 alters the H3K4 dimethylation and H3 acetylation profiles, and the localization of histone deacetylase 3, at *IRF1*. While MEN1 knockdown does not impact the rate of transcription, *IRF1 *heteronuclear transcripts become enriched in MEN1-depleted cells. The processed mRNA and translated protein product are concomitantly reduced, and the antiviral state is attenuated. Additionally, the transcription start site at the *IRF1 *promoter is disrupted in the MEN1-depleted cells. The H3K4 demethylase, lysine specific demethylase 1, is also associated with *IRF1*, and its inhibition alters H3K4 methylation and disrupts the transcription start site as well.

**Conclusions:**

Taken together, the data indicate that MEN1 contributes to STAT1-activated gene expression in a novel manner that includes defining the transcription start site and RNA processing.

## Background

Eukaryotic gene expression is regulated by dynamic nuclear signaling events that occur at the chromatin template and include post-translational modification of the histone proteins, via methylation, phosphorylation, acetylation and ubiquitination. Depending upon the position of a lysine residue in the histone amino acid sequence, histone lysine methylation is associated with either an activated or a repressed transcriptional state [1,2]. For example, methylation of K27 and K9 of histone H3 and K20 of histone H4 correlates with transcriptionally silent regions, while methylation of K4, K36 and K79 of histone H3 is associated with activated chromatin. Lysine methylation is further elaborated by the degree of methylation, such that mono-, di- or trimethylation of the same lysine residue can affect chromatin structure differently.

In *Saccharomyces cerevisiae*, a single complex containing the histone methyltransferase (HMT) Set1 is responsible for the methylation of histone H3K4. In mammalian systems, six homologs of Set1 contribute to different Set1-like HMT complexes to accomplish this modification [1,2]. The Set1A/Set1B complexes are most similar to yeast Set1, and reportedly drive the majority of the H3K4 methylation in mammalian cells [3]. The HMT activity of the four other Set1-like complexes derives from the mixed lineage leukemia (MLL) family of proteins (MLL1 to MLL4), but how their gene targets are specified is not known [4]. All the human Set1-like complexes share a quartet of proteins, absent, small, homeobox-like Drosophila (ASH2L), retinoblastoma binding protein 5 (RbP5), WD repeat domain 5 (WDR5) and human dosage compensation-related protein (hDPY-30) [5], but the interacting proteins menin (MEN1) and pax transactivation domain-interacting protein (PTIP) are respectively specific to the MLL1/MLL2 and the MLL3/MLL4 complexes.

Menin (MEN1) is the product of the tumor suppressor gene *MEN1 *that is mutated in the inherited syndrome multiple endocrine neoplasia type 1. MEN1 is predominately localized to the nucleus, and is reported to associate with several transcription factors to both repress (JunD, NF-kB) and stimulate (Smad3, ERα, VDR, PPARγ) gene activation [6]. In addition, MEN1 impacts the cell cycle by promoting the expression of cyclin-dependent inhibitors, p18^INK4c ^and p27^Kip1 ^[7,8]. Since H3K4 methylation is generally thought to positively affect transcription, and MEN1 is a component of the Set1-like MLL1/MLL2 complexes, MEN1's role in transcriptional activation is thought to reflect the proper recruitment of a co-activating H3K4 methyltransferase to particular gene promoters [9].

There is, however, some evidence that H3K4 methylation can be repressive of transcription, in a manner that is similar to the repressive function of the histone modification made by Set2, namely H3K36 methylation. There, a histone deacetylase (HDAC) complex - Rpd3C(S), that can recognize H3K36 methylation states - deacetylates histones within transcribed regions to prevent transcription from cryptic promoters located in the 3' end of genes [10]. Similarly, the mSIN3a-HDAC1 complex is recruited by H3K4 trimethylation (H3K4me3) to repress the cyclin D gene [11,12], and two distinct HDAC complexes, Set3-HDAC (Set3C) and Rpd3C(S), recognize H3K4 dimethylation (H3K4me2). In the case of Set3C, HDAC recruitment is to the 5'-end of actively transcribed genes, and it promotes efficient transcription [13]. In the case of Rpd3C(S), H3K4me2 is established by cryptic transcription that begins upstream of the promoter, and it is used to maintain an HDAC activity to attenuate transcription from the normal promoter (at *GAL1*) or from a hidden promoter (at *SUC2*) [14]. Set1 activity also represses TY1 transposon transcription and mobility in a mechanism that, again, depends upon HDAC activity [15].

In response to external signals, the signal transducers and activators of transcription (STAT) family of transcription factors activates gene expression to promote cellular growth, differentiation, homeostasis, inflammation and the immune response [16]. *Interferon regulatory factor 1 *(*IRF1*) is a primary interferon (IFN) response gene that is highly induced by IFN-γ, and whose transcription is mediated by a STAT1 DNA binding element [17]. STAT1-activated gene expression of *IRF1 *is rapid and transient, and requires dynamic post-translational modification of the chromatin template, including H3K4 methylation [18]. H3K4me3 is found at the promoter of *IRF1*, and increases when this gene is activated by IFN-γ treatment. H3K4me2 is localized to the 5' region of this gene, in both the induced and uninduced states, and in cells null for STAT1 as well. H3K4me2, therefore, is maintained via a mechanism that does not require STAT1 triggered transcriptional activation of *IRF1*. Inhibition of H3K4 HMT activity decreases STAT1 activated gene expression and prevents the inducible H3K4me3. MEN1 is associated with the *IRF1 *gene locus, and is transiently recruited or stabilized during IFN-γ induction of STAT1 signaling.

To more fully study MEN1's function at *IRF1 *during the rapid and transient gene expression invoked downstream of STAT1 activation, we generated a cell line where MEN1 is depleted. The collected data point to a mechanism for MEN1 that is more complicated than its previously described role in regulating activated transcription [19-21]. Instead, the results suggest that MEN1 and H3K4me2 function to maintain the fidelity of the *IRF1 *core promoter transcriptional start site and a chromatin environment that efficiently promotes mRNA processing.

## Results

### Characterization of MEN1 microRNA-adapted shRNA (shRNAmir) and non-silencing shRNAmir cell lines

**Figure 1:**
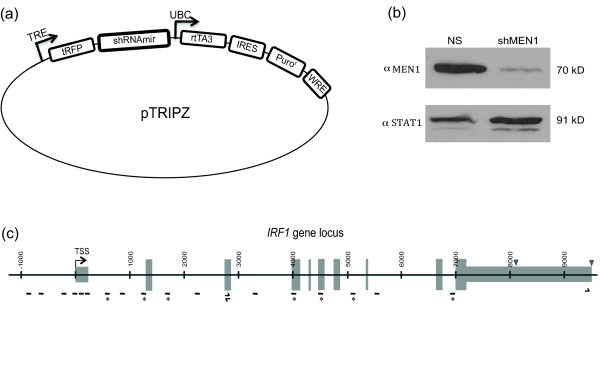
**Characterization of *MEN1-shRNAmir *and non-silencing *shRNAmir *cell lines. (a) **Graphic depiction of the pTRIPZ shRNAmir vector. The pTRIPZ transactivator, reverse tetracycline transactivator 3 (rtTA3), binds to and activates expression from a TRE promoter in the presence of doxycycline, for the inducible expression of an shRNAmir. **(b) **Western blotting of whole cell extracts from a stable 2fTGH cell line either expressing the inducible knockdown shRNAmir vector targeting MEN1 (shMEN1) or a nonsilencing shRNAmir (NS) vector were subjected to SDS-PAGE, blotted on nitrocellulose and a MEN1 antibody was used to develop the blots. STAT1 served as a loading control. Quantification was performed using Image J. **(c) **Graphic depiction of the *IRF1 *gene locus (approximately 9 kb) indicating the TSS, location of the two polyadenylation signals (inverted triangles) and locations of ChIP qPCR primers (dashes) and RACE primers (arrows) used in this study. * indicates primers also used in qRT-PCR. ChIP: chromatin immunoprecipitation; kb: kilobases; MEN1: multiple endocrine neoplasia type 1; qPCR: quantitative PCR; qRT-PCR: quantitative real time PCR; RACE: rapid amplification of cDNA ends; STAT1: signal transducer and activator of transcription 1; TSS: transcription start site.

To determine MEN1's mechanism at *IRF1 *during STAT1-activated transcription, we generated a stable 2fTGH cell line where MEN1 is depleted using the inducible knockdown shRNAmir vector pTRIPZ (Open Biosystems, Huntsville AL USA, Figure 1a). A control 2fTGH cell line stably expressing a non-silencing shRNAmir was selected as well. Quantification of western blots of cell extracts from these two cell lines consistently showed that endogenous MEN1 is reduced by approximately 95% in the *shRNAmir-MEN1 *cell line (Figure 1b). A second constitutive pGIPZ shRNAmir vector targeting MEN1 was as effective for knockdown and a stable cell line expressing this vector showed the same transcriptional defects described below (data not shown). A schematic of the *IRF1 *gene locus is shown in Figure 1c.

### The H3K4me2 profile at *IRF1 *is altered by MEN1 depletion

**Figure 2:**
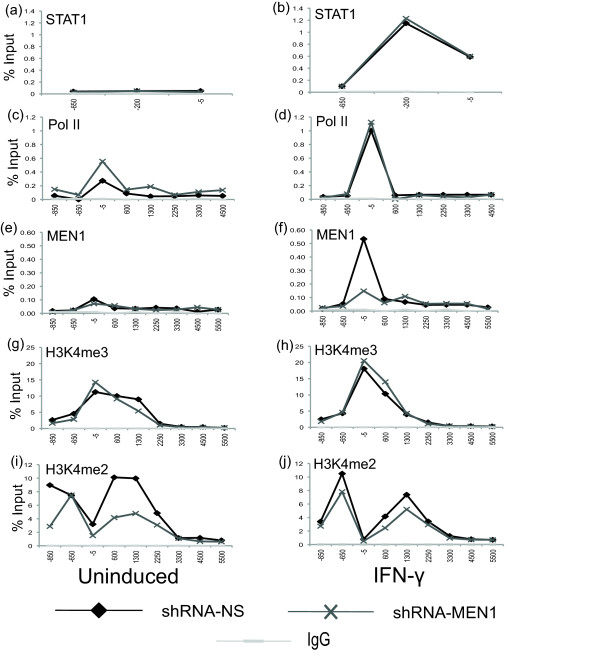
**The H3K4 dimethylation profile at *IRF1 *is altered by MEN1 depletion. (a-j) **Crosslinked chromatin immunoprecipitation of *shRNAmir-MEN1 *(shRNA-MEN1) and *shRNAmir-nonsilencing *(shRNA-NS) cell lines treated with IFN-γ for 30 minutes or uninduced. The indicated antibodies were used and qPCR quantified the precipitate yield using primers spanning the *IRF1 *locus (see Figure 1c), reported as percent of input. Immunoglobulin G served as the negative control. *P *≤ 0.05 for 2f, i. IFN: interferon; qPCR: quantitative PCR; MEN1: multiple endocrine neoplasia type 1.

Previously, we profiled H3K4me2 and -me3 in response to IFN-γ induction of STAT1 signaling at the *IRF1 *gene, using chromatin immunoprecipitation (ChIP) [18]. These modifications, as well as STAT1, RNA polymerase II (Pol II) and MEN1, were assayed in the *shRNAmir-MEN1 *cell line and in the control cell line before and after treatment with IFN-γ (Figure 2). STAT1 is normally recruited to its DNA binding site (near -200 bp), as is Pol II to the transcription start site (TSS) in MEN1-depleted cells (Figure 2a, b, c, d). MEN1, as expected, is not localized to the promoter in response to IFN-γ in the *shRNAmir-MEN1 *cell line (Figure 2f), but is recruited normally in the control cells. Surprisingly, the profile for H3K4me3 does not appear to be altered by MEN1 depletion in either the uninduced or induced condition (Figure 2g, h). On the other hand, the H3K4me2 profile is two-and-a-half times lower in the uninduced state in the *shRNAmir-MEN1 *cell line, such that the H3K4me2 level does not change when the cells are induced with IFN-γ, as it does in the control cell line (Figure 2i, j). The differences in the H3K4me2 ChIP signal normally observed during induction most likely reflect either a conversion from the dimethyl to the trimethyl state at K4 or the recognition of the H3K4me2 moiety by another protein, or both.

### Histone H3 acetylation and HDAC3 localization are altered in MEN1-depleted cells

The difference in H3K4me2, but not H3K4me3 in the *shRNAmir-MEN1 *cell line suggested that, as in *S. cerevisiae*, two distinct chromatin environments might be associated with these modifications [13]. In yeast, H3K4me3 reportedly promotes high acetylation levels and histone depletion at promoters, while H3K4me2 can recruit HDACs to suppress acetylation. During activated transcription, the HDAC complex Set3C deacetylates downstream of the promoter to facilitate transcription [13], while the Rpd3C(S) HDAC complex functions in the transcriptionally inactive state to suppress promoter activity [14].

**Figure 3:**
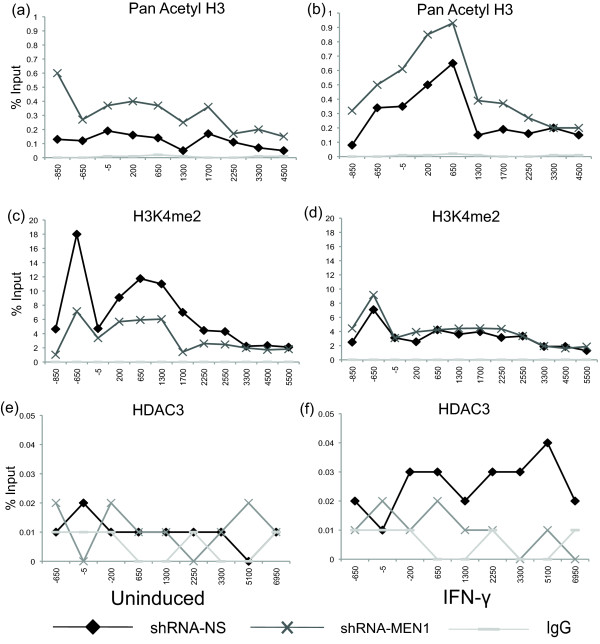
**Histone H3 acetylation and HDAC3 localization are altered in MEN1-depleted cells. (a-d) **Micrococcal nuclease ChIP of shRNA-MEN1 and shRNA-NS cell lines treated with IFN-γ for 30 minutes or uninduced. The indicated antibodies were used and qPCR quantified the precipitate yield, reported as percent of input. **(e, f) **Crosslinked ChIP of shRNA-MEN1 and shRNA-NS cell lines treated with IFN-γ for 30 minutes or uninduced. HDAC3 antibody was used and qPCR quantified the precipitate yield, reported as percent of input. Immunoglobulin G served as the negative control. *P *≤ 0.05 for 3a, b, c, f. ChIP: chromatin immunoprecipitation; HDAC: histone deacetylase; IFN: interferon; MEN1: multiple endocrine neoplasia type 1; NS: nonsilencing; qPCR: quantitative PCR.

First, to determine if there was a deacetylation defect associated with the observed loss of H3K4me2 in the MEN1-depleted cell line, we performed ChIP assays for histone H3 acetylation. In order to achieve higher resolution in the ChIP assay, cells were not fixed with formaldehyde, and the chromatin was digested with micrococcal nuclease rather than sheared via sonication. In both the uninduced and induced states, significantly more (approximately three-fold) histone H3 acetylation was immunoprecipitated from the *shRNAmir-MEN1 *cell line than from the non-silencing shRNAmir control cell line (Figure 3a, b). The H3K4me2 profiles were essentially the same here (Figure 3c, d) as in the cross-linked ChIP assay (Figure 2). These data suggested that an HDAC activity might be lost or impaired in the *shRNAmir-MEN1 *cell line, where H3K4me2 is low.

It is well established that an HDAC activity is required for IFN-stimulated gene expression [22-24], and Nusinzon and Horvath showed that it is HDAC1 that promotes IFN-αinduced transcription [23]. IFN-γ induced transcription shares the same HDAC requirement, and HDAC1 physically interacts with STAT1, though whether it is the HDAC activity needed is not explicitly known [23,24]. Moreover, MEN1 has been reported to associate with an mSin3a-HDAC1 complex for the repression of JunD transcription [25]. Therefore, we initially attempted to ChIP HDAC1 at the *IRF1 *gene locus but were unable to detect any enrichment above the negative control (immunoglobulin G (IgG)) levels with nine different primer sets that spanned the region -450 bp to +7000 bp (data not shown). A control quantitative PCR primer set, flanking an Sp1 binding site in the human p21 promoter and provided by the antibody manufacturer for use as a positive control, did show some HDAC1 enrichment (data not shown).

Since HDAC3 is the closest mammalian homolog to yeast Hos2, one of the histone deacetylases that contributes to the Set3 complex that recognizes H3K4me2 [13,26], we also performed a ChIP assay that targeted HDAC3. HDAC3 was ChIP'd but no difference in its enrichment between the control and knockdown cell lines was observed in the uninduced state. However, when the *shRNAmir-MEN1 *and non-silencing control cell lines were induced with IFN-γ, the HDAC3 levels increased three- to four-fold in the control cell line, but not in the MEN1 knockdown cell line (Figure 3e, f). Taken together, these data suggest that H3K4me2 might provide a docking site for a complex containing HDAC3, in order to oppose the histone H3 acetylation that occurs at *IRF1 *in response to IFN-γ. We attempted to ChIP other components of a putative HDAC3 complex, specifically the silencing mediator of retinoid and thyroid receptors (SMRT) and the nuclear receptor corepressor (NCoR) [13,27]. However, ChIP signals that were significantly different from the negative control were not observed using SMRT or NCoR antibodies, at any position across the *IRF1 *locus (data not shown).

### *IRF1 *heteronuclear RNA (hnRNA) transcripts are enriched in the MEN1-depleted cell line

To determine if transcription of the *IRF1 *gene was affected by depletion of MEN1, we performed quantitative reverse transcription PCR (qRT-PCR). Using primer pairs that are complementary to both intronic and exonic *IRF1 *sequences, the relative abundance of *IRF1 *transcripts in the hnRNA and total RNA pools was determined for the *shRNAmir-MEN1 *and non-silencing control cell lines, in the uninduced and induced states.

**Figure 4:**
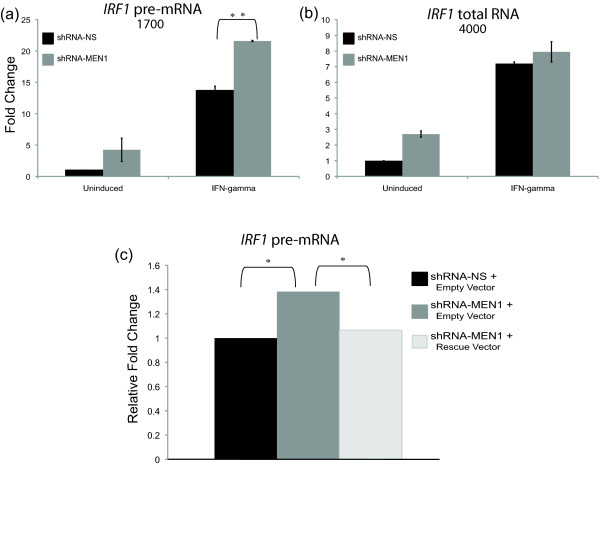
***IRF1 *hnRNA transcripts are enriched in the MEN1-depleted cell line. (a, b) **qRT-PCR to quantitate *IRF1 *mRNA and pre-mRNA expression in shRNA-MEN1 and shRNA-NS cell lines that were uninduced or treated with IFN-γ. *IRF1 *expression was normalized to *β-actin *and fold change relative to the uninduced shRNA-NS condition was calculated. Error bars are standard error (n = 4). **P ≤ 0.01, *P ≤ 0.05. Numbers are the base pair location of the primers used on the *IRF1 *gene; see Figure 1c. **(c) **qRT-PCR to quantitate *IRF1 *pre-mRNA expression in shRNA-MEN1 cells transiently transfected with a plasmid expressing MEN1 or an empty vector. *IRF1 *expression, normalized to *β-actin *and fold change calculated as above, is presented relative to shRNA-NS cells (black bar) transiently transfected with empty vector and induced. Student's t-test determined significance. *P ≤ 0.05. IFN: interferon; MEN1: multiple endocrine neoplasia type 1; NS: nonsilencing; qRT-PCR: quantitative real time PCR.

Consistently, we observed an approximately two-fold increase in the number of *IRF1 *transcripts populating the hnRNA pool in the IFN-γ induced MEN1-depleted cell line as compared to the control cell line, while the *IRF1 *transcripts contributing to total RNA (exonic) were indistinguishable between the two cell lines (Figure 4a, b). This result was rescued when the MEN1 knockdown was reversed by overexpression of a *MEN1 *cDNA (Figure 4c), and was reproduced using four additional intronic primer pairs and one additional exonic primer pair that span *IRF1 *(Additional File 1).

hnRNAs are typically short-lived, and their relative quantities correlate with transcription rates so that qRT-PCR using intronic primer sets can be used as a surrogate for the more cumbersome and less sensitive nuclear run-off assay [28]. However, the enrichment in hnRNA *IRF1 *transcripts in the MEN1-depleted cells as compared to the control cells is not observed earlier in IFN-γ induction (Additional File 2), suggesting that the increase in *hnIRF1 *is not simply due to an increased rate of transcription.

### *IRF1 *mRNA becomes under-represented in MEN1 knockdown cells

**Figure 5:**
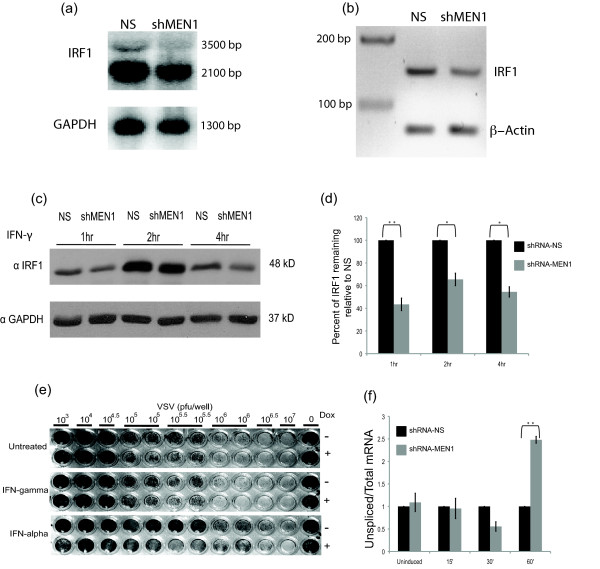
***IRF1 *mRNA becomes underrepresented in MEN1 knockdown cells. (a) **Northern blot analysis of RNA collected from shRNA-MEN1 (shMEN1) and shRNA-NS (NS) cells, induced with IFN-γ for 2 hours. The blot was probed with ^32^P-labeled *IRF1 *and *GAPDH *(loading control) cDNA probes. Quantification was done with the Storm 840 Imager and ImageQuant TL. **(b) **RT-PCR of RNA collected from shRNA-MEN1 and shRNA-NS cells, using primers designed to exon 4 (forward) and exon 5 (reverse), to specifically amplify *IRF1 *mRNA. *β-actin *primers were used as a loading control. **(c) **Western blot of extracts prepared from shRNA-MEN1 and shRNA-NS cells uninduced or induced with IFN-γ for indicated times. An IRF1 antibody was used to develop the blots. GAPDH served as a loading control. **(d) **Graphical representation of western blots (n = 3) as in panel C. IRF1 protein levels in the two cell lines were first normalized to GAPDH and then shRNA-MEN1 IRF1 levels were compared to shRNA-NS IRF1 levels. Quantification was performed using Image J. Error bars are standard error. **(e) **Cytopathic effect of VSV in the *shRNAmir-MEN1 *cell line. Cells were cultured with (+) or without (-) doxycycline, treated with IFN-γ, IFN-α or left untreated for 12 hours and infected with dilutions of VSV. One of two biological replicates is shown. **(f) **qRT-PCR to detect the ratio of unspliced to total *IRF1 *RNA at the indicated times after IFN-γ induction (n = 4). The shRNA-MEN1 ratio is presented in reference to the shRNA-NS ratio, which was set to 1 at each condition. Error bars are standard error. **P ≤ 0.01, *P ≤ 0.05. IFN: interferon; MEN1: multiple endocrine neoplasia type 1; NS: nonsilencing; RT-PCR: real time PCR; VSV: vesicular stomatitis virus.

An alternative explanation for the qRT-PCR results is that the processed *IRF1 *mRNA species becomes under-represented in the MEN1 knockdown cell line. To show this explicitly, northern blotting was performed using *IRF1 *cDNA as a probe (Figure 5a). *IRF1 *is alternatively polyadenylated, and quantification of the predicted 2.1 kb *IRF1 *mRNA transcript and the larger 3.5 kb mRNA transcript (Figure 1c) induced by IFN-γ shows 47% fewer of these RNA species in the MEN1 knockdown cell line. The same result was observed in an RT-PCR reaction designed to specifically amplify the mRNA species (Figure 5b). The predicted PCR product of 120 bp is 54% lower in the MEN1 knockdown cell line. Larger RNA species that contain intronic sequences detectable via qRT-PCR (Figure 4) were not observed via northern blotting or RT-PCR. Since protein expression levels typically reflect mRNA expression levels, we also asked if IRF1 expression was lower in the *shRNAmir-MEN1 *cells as compared to the non-silencing control cells (Figure 5c). In three separate experiments, western blotting showed an average 50% loss of IRF1 protein in the induced *shRNAmir-MEN1 *cells, from one hour to four hours post IFN-γ induction (Figure 5d).

The decrease in IRF1 expression suggested that the antiviral response induced by IFNs might be attenuated in the *shRNAmir-MEN1 *cell line [29]. To test this idea, the *shRNAmir-MEN1 *cell line was analyzed in a cytopathic effect (CPE) assay as described in [30] (Figure 5e). *shRNAmir-MEN1 *cells were cultured with and without doxycycline, treated with IFN-γ IFN-αor left untreated, then infected with dilutions of vesicular stomatitis virus (VSV). Western blotting showed that doxycycline induced a 60% loss of MEN1 expression (Additional File 3). In the untreated condition, there was no difference in the CPE. However, with both IFN-γ and IFN-α, the MEN1-depleted cells were less effective at establishing an antiviral state in this assay. The modest size of the effect likely reflects the fact that IRF1 protein expression is only partially diminished in the *shRNAmir-MEN1 *cell line. It is similar to the effect observed for the STAT1 mutant, (STAT1 S727A), where a single serine-to-alanine mutation also causes a partial loss of its IFN-γ induced transcriptional activity [30].

The concomitant increase in *IRF1 *hnRNA transcripts and decrease in *IRF1 *mRNA suggested that MEN1 depletion could contribute to a defect in splicing efficiency. Indeed, previous research has revealed a link between histone H3K4 methylation and splicing; H3K4me3 is recognized by chromodomain helicase DNA-binding protein 1 for the recruitment of spliceosomal components of the U2 snRNP to enhance the rate of the splicing reaction at *IRF1 *[31]. In that study, a delay in the splicing efficiency was observed early in *IRF1 *induction, but it resolved quickly. When we determined the *IRF1 *splicing efficiency in the same way, it appeared to proceed normally until one hour post-induction (Figure 5f), suggesting a different mechanistic defect in mRNA splicing occurs when MEN1 is knocked down. Unfortunately, attempts to ChIP the splicing factor 3a complex subunits were unsuccessful. Since the C-terminal domain (CTD) of Pol II is involved the recruitment of splicing complexes [32], we assayed for alterations in the phosphorylation of the CTD of Pol II using ChIP antibodies that recognize total Pol II and serine 2 phosphorylation in the CTD, but found no significant differences between the knockdown and control cell lines (Additional File 4).

### The *IRF1 *transcription start site (TSS) is disrupted in MEN1-depleted cells

Mutation of both *SET2 *and the unique components of the Rpd3C(S) complex - *RCO1 *and *EAF3 *- cause an accumulation of aberrant transcripts starting from cryptic, intragenic promoters found in the 3'-ends of genes [33-35]. Similarly, suppression of spurious transcription in the 5'-end of genes is a function postulated for the yeast Set1/Set3C pathway, but such cryptic transcripts have not been reported [13].

**Figure 6:**
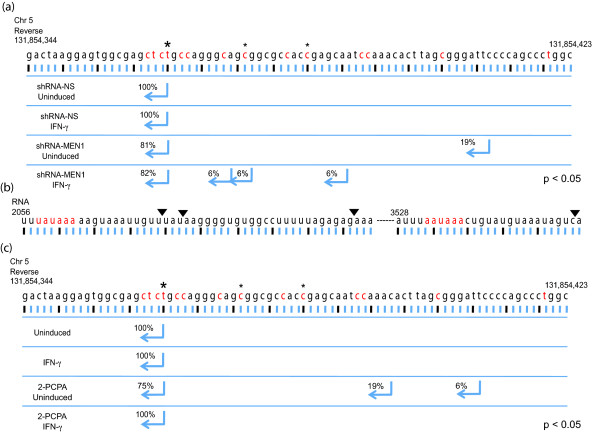
**The *IRF1 *TSS is disrupted in MEN1-depleted cells. (a) **5' RACE-PCR of RNA collected from shRNA-MEN1 and shRNA-NS cells, with or without treatment with IFN-γ. PCR products were generated using the 5' RACE primer along with a reverse primer designed to the third exon of *IRF1*. Arrows indicate the base position on the reverse strand of chromosome 5 to which cDNA ends mapped and the percentages reflect the frequency that a site was observed. Asterisks mark the position of major CTSSs determined in [39] with the large asterisk indicating the RefSeq TSS. Red indicates other minor CTSSs. 2fTGH cells, +/- IFN-γ data were the same as the shRNA-NS data. **(b) **3' RACE-PCR products were generated using a polydT primer along with a forward primer designed to the third exon or the tenth exon of *IRF1*. Inverted triangles indicate observed cleavage sites on the *IRF1 *mRNA. Poly(A) signal sequences are in red. **(c) **5' RACE-PCR of 2-PCPA treated and untreated cells, with or without IFN-γ induction. Primers are depicted in Figure 1c. 2-PCPA: trans-2-phenylcyclopropylamine; CTSS: cap analysis of gene expression tag starting site; IFN: interferon; MEN1: multiple endocrine neoplasia type 1; NS: nonsilencing; PCR: polymerase chain reaction; RACE: rapid amplification of cDNA ends; TSS: transcription start site.

To determine if 5' cryptic transcripts accumulate from the *IRF1 *gene in the *shRNAmir-MEN1 *cell line, we performed 5' rapid amplification of cDNA ends (RACE)-PCR assays with mRNA isolated from the *MEN1*-depleted cells and control cells. In both the uninduced and induced states, approximately 20% of the transcripts in *shRNAmir-MEN1 *cells began at nucleotides that map nine to forty-seven base pairs upstream of the canonical TSSs, as defined by the RefSeq mRNA collection [36]. Of the *IRF1 *transcripts characterized from the non-silencing shRNA control cells (Figure 6a) and wild-type 2fTGH cells, 100% started at the RefSeq TSS. Importantly, all the 5'RACE-PCR products that were purified, cloned and sequenced showed proper splicing.

We attempted to confirm the 5' RACE-PCR data using primer extension analysis, but found that this approach was not sufficiently sensitive for the detection of the aberrant transcripts characterized via 5' RACE-PCR. Therefore, we developed a qRT-PCR strategy to quantify transcripts that started upstream of the canonical TSS for *IRF1*. To determine if these aberrant transcripts were unique to the MEN1-dependent gene *IRF1*, we employed the same strategy to detect similarly aberrant transcripts at another Pol II gene that does not depend upon MEN1, namely *GAPDH*, and at the Pol I and Pol III genes, *RN18S1 *[37] and *RN7SK *[38] (Additional File 5). Aberrant TSS *IRF1 *transcripts were detected using RNA extracted from both the *shRNAmir-MEN1 *and *shRNAmir-NS *cell lines. However, an average three-and-a-half-fold enrichment in these aberrant TSS transcripts was observed when MEN1 was depleted. For *GAPDH*, aberrant TSS transcripts were detected as well, but not to the same extent in both the *shRNAmir-MEN1 *and *shRNAmir-NS *cell lines. No aberrant TSS transcripts were detected for *RN18S1 *and *RN7SK*.

The cap analysis of gene expression (CAGE) viewer for *Homo sapiens *(hg17; http://gerg01.gsc.riken.jp/cage/hg17prmtr/) was consulted to see if any of the alternative TSSs discovered here were also found in this genome-wide analysis of mammalian promoter structure [39]. At the *IRF1 *gene promoter, a total of 86 tags were mapped to 15 CAGE tag starting sites (CTSSs): 47% of the tags mapped to the RefSeq TSS defined as +1; 15% mapped to the base at -20 relative to the TSS, and 10% mapped to the base at -11. The remaining tags represented < 1% to 6% of the total tags. In the CAGE study, 58.6% of tags had a pyrimidine-purine dinucleotide at positions -1, +1, suggesting a preference for transcriptional initiation from this sequence. All three of the major CTSSs at +1, -11 and -20 possess the pyrimidine-purine dinucleotides at positions -1, +1. None of the alternate TSSs we defined have this dinucleotide sequence pattern.

We also determined the 3'-end of the *IRF1 *transcripts via 3' RACE-PCR (Figure 6b). The *IRF1 *mRNA is alternatively polyadenylated, using polyadenylation signals approximately 2060 and 3530 bases downstream of its 5'-end [36,40]. When using a primer to exon 3, all of the transcripts characterized from both cell lines used the polyadenylation signal (UAUAAA), nearly identical to the consensus signal (AAUAAA), at approximately base 2060. Although there were some differences in the precise cleavage site used, these differences were seen in both the control and knockdown cell lines. The longer *IRF1 *mRNA species is less prevalent, so a primer from exon 10 was used to generate 3' RACE-PCR products containing the RefSeq mRNA polyadenylation signal (AAUAAA) near base 3530. All the 3' RACE-PCR products that were sequenced here showed that the mRNA was cleaved at a single site. Regardless of the primer used, all the 3' RACE-PCR products were of the size predicted by the spliced mRNA. Thus, there was no evidence of polyadenylated RNAs that were unspliced. We conclude that polyadenylation occurs normally in both the MEN1 knockdown and control cells.

Taken together, these data indicate that the *IRF1 *TSS is less constrained in the cell line where MEN1 is depleted and, more significantly, that *IRF1 *transcripts are not processed as efficiently, though it is not necessarily the case that these two transcriptional defects are coupled.

### Lysine specific histone demethylase 1 isoform a (LSD1) is associated with the *IRF1 *gene and its inhibition also disrupts the basal TSS

**Figure 7:**
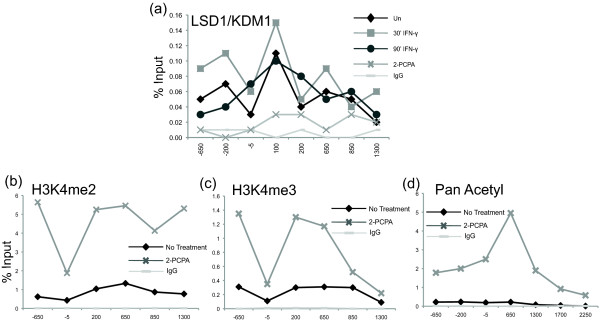
**Chemical inhibition of LSD1 increases acetylation and H3K4 methylation at the *IRF1 *gene. (a) **ChIP of 2-PCPA treated cells and control cell lines treated with IFN-γ for 30 minutes and 90 minutes or uninduced and collected at the times indicated. An LSD1 antibody was used and qPCR quantified the precipitate yield, reported as percent of input. **(b-d) **ChIP of 2-PCPA treated and untreated cell lines. The indicated antibodies were used and qPCR quantified the precipitate yield, reported as percent of input. Immunoglobulin G served as the negative control. *P *≤ 0.05 for 7b, c, d and crosses versus black diamonds in 7a. 2-PCPA: trans-2-phenylcyclopropylamine; ChIP: chromatin immunoprecipitation; IFN: interferon; LSD1: lysine specific histone demethylase 1 isoform a; qPCR: quantitative PCR.

Interestingly, we observed the same loss of fidelity at the *IRF1 *TSS in another experimental system where H3K4me2 levels are modulated. Lysine specific histone demethylase 1 isoform a (LSD1, also known as KDM1a) is a histone H3K4 demethylase [41] that is associated with the *IRF1 *promoter in both uninduced and induced states (Figure 7a). Treatment with the LSD1-specific inhibitor trans-2-phenylcyclopropylamine (2-PCPA) appeared to remove LSD1 from the *IRF1 *promoter (Figure 7a). This result might be due to the fact that 2-PCPA covalently modifies LSD1, and this prevents LSD1 from binding at the promoter [42]. Alternatively, the covalent adduct might interfere with antibody recognition. Regardless, as would be predicted, LSD1 inhibition with 2-PCPA resulted in approximately four-fold increases in both H3K4me2 and H3K4me3 (Figure 7b, c). LSD1 is found in complex with its cofactor, RE1-silencing transcription factor co-repressor (CoREST), and the histone deacetylases HDAC1 and 2 [41], and there is an intimate functional relationship between LSD1 and HDAC1. Both enzymes require the cofactor CoREST and enzymatically active LSD1 is required for efficient deacetylation by HDAC1 [42,43]. This predicted that the *IRF1 *promoter would be hyperacetylated in the uninduced 2-PCPA-treated condition. ChIP using a pan acetyl H3 antibody confirmed this was the case; this ChIP signal was up to 25-fold higher when cells were treated with 2-PCPA (Figure 7d). When 5' RACE-PCR was performed on cells treated with 2-PCPA, 25% of the RACE-PCR products began upstream of the canonical TSS (Figure 6c). Induction with IFN-γ however, appeared to re-establish the proper TSS.

When taken together, the data suggest that hyperacetylation of the *IRF1 *promoter, whether due to loss of H3K4me2 as a result of MEN1 depletion, or LSD1-CoREST-HDAC1 inhibition, results in fewer structural constraints in the chromatin template for defining the canonical TSS.

## Discussion

### Histone modification and transcriptional fidelity

In this report, we demonstrate that during STAT1-triggered gene activation, depletion of the Set1-like MLL1/MLL2 complex component MEN1 is associated with disruption of the TSS at the *IRF1 *core promoter, and with diminished processing of the *IRF1 *hnRNA transcript to the mature mRNA. Depletion of MEN1 correlates with decreased H3K4me2 at the *IRF1 *promoter, increased H3 acetylation, and loss of HDAC3 recruitment in response IFN-γ induction. Moreover, IRF1 protein levels are accordingly lower when MEN1 is knocked down, resulting in an attenuated antiviral response in this cell line.

There are several possible explanations for the defects in transcriptional fidelity observed here. For instance, it is well established that the rate of elongation by Pol II [43,44], the recruitment of splicing factors by its CTD [44], and the core promoter itself [45] can all regulate alternative pre-mRNA splicing. While *IRF1 *is not alternatively spliced, and a more general deficiency in intron processing is likely, it remains possible that these functions are distorted in the MEN1 knockdown cell line, as experiments to address some of them yielded negative results.

Perhaps more significant though is the recent but growing evidence that chromatin structure can impact the regulation of splicing, giving credence to the idea that histone modifications themselves function in the regulation of co-transcriptional RNA-processing events [46,47]. Thus, based on the results of this study, we propose a working hypothesis in which MEN1, as part of a Set1-like HMTase complex that targets H3K4 for dimethylation, establishes and maintains a chromatin environment - via HDAC3 recruitment - that promotes transcriptional fidelity during ongoing rounds of transcription. Determining the precise mechanism by which MEN1 achieves this proper chromatin environment requires further study and the development of new experimental tools, effective ChIP-grade antibodies in particular. However, these observations are somewhat akin to those reported in yeast where the H3K4me2 modification is recognized by the Set3 complex that deacetylates histones in 5' transcribed regions [13]. Both Set3 and Hos2 were shown to be required for efficient activation of *GAL1 *[26], and Pol II's interaction at this gene was disrupted by *SET3 *deletion [13]. This is different from the results reported here, in that *IRF1 *activation was unaffected by MEN1 depletion, and no differences in Pol II interaction with *IRF1 *were observed. Rather, we speculate that it is processing to the mRNA that is diminished as transcription continues, a process that is not relevant at the yeast *GAL1 *gene.

More recent support for our working hypothesis comes from a report by Gunderson *et al. *in which dynamic acetylation (via Gcn5) and deacetylation (via Hos2/3) at intron-containing genes in yeast was found to affect co-transcriptional splicing by promoting dynamic rearrangements of the spliceosome [48]. In this study, improperly persistent acetylation stabilized interactions that are meant to be transient, slowing the release of U2 snRNP and thereby slowing co-transcriptional RNA processing. Thus, the chromatin-based co-transcriptional defect described in this study is very similar to the one we propose.

### Histone modification and TSS fidelity

In a 2009 review of studies of H3K4 dimethylation in yeast [49], Pinskaya and Morillon asked whether H3K4me2-HDAC mechanisms might be conserved in higher eukaryotes. The data reported here are the first indication that they are. In addition, these authors presented the idea that H3K4me2 might be part of a signaling pathway meant to ensure promoter fidelity, and suggested three potential models involving a recruited HDAC and the RNA degradation complex Nrd1/Nab3. A similar idea was put forward by Kim and Buratowski in considering the results of their study on the Set1-Set3C pathway [13]. Both propose that an HDAC activity is recruited to H3K4me2 at the promoter to remove acetylation on adjacent nucleosomes, in order to prevent Pol II from initiating at uncovered TSSs. However, the predicted 5' cryptic TSSs were not found in these yeast studies. In contrast, in our study, several alternative TSSs at *IRF1 *were characterized when H3K4me2 levels were experimentally modulated. Thus, this is the first direct evidence of an H3K4me2-HDAC mechanism in maintaining eukaryotic TSS fidelity by Pol II. In future studies, the hypothesis that H3K4me2 recruits a particular HDAC complex in the uninduced state will be addressed. We suspect that once transcription is induced, TSS fidelity comes to depend less on how the chromatin architecture was established in the uninduced state. This might explain why it was difficult to detect RNA transcripts from alternative TSSs, even after IFN-γ induction.

### A novel role for MEN1 in activated gene expression

The results of this study suggest a different and more complicated function for MEN1 in STAT1-activated transcription than has been reported thus far for MEN1 during the inducible expression of other genes. As examples, three studies of nuclear receptor (NR)-mediated transcriptional activation have described a co-activating role for MEN1. MEN1 directly interacts with ERα and PPARγ, and acts as a co-activator during their ligand activated transcription [19,20]. MEN1 can also co-activate VDR mediated transcription [21]. In these cases, loss of MEN1 leads to decreased expression of NR target gene mRNAs (as measured by qRT-PCR), in response to NR ligand addition, and lower H3K4me3 at these genes' promoters. Thus, MEN1's role in transcriptional activation is understood to simply reflect the proper recruitment of a co-activating methyltransferase to gene promoters for the methylation of histone H3 at K4 [9].

We do not observe any change in the enrichment of H3K4me3 at the *IRF1 *promoter when MEN1 is depleted. Nor do we observe any loss in total *IRF1 *RNA levels. These dissimilar observations might reflect differences in the kinetics of gene activation by NRs versus STATs. But, it is also possible that STAT1-activated gene expression requires regulation from the chromatin environment that is distinct from that needed during NR-activated gene expression, and that MEN1 contributes by establishing and maintaining the necessary levels of H3K4me2 at STAT1-dependent promoters. The same explanation is likely true when comparing our results to those reported at the constitutive *HOXA9 *locus [50]. There, *MEN1 *excision correlates with a two-fold loss of H3K4me3. Going forward, it will be important to tease apart if or how the Set1-like MLL1/MLL2 complexes, of which MEN1 is a component, specifically generate the sharp peak of H3K4 trimethylated histones versus the broader peak of dimethylated histones at the 5' end of genes, since these two methyl marks appear to foster the opposing activities of acetylation and deacetylation. And it will be interesting to determine how the different functions of the Set1-like MLL1/MLL2 complexes are specified at different genes. In other words, when is the straightforward co-activating function, requiring H3K4me3, delivered as opposed to the more complicated H3K4me2-HDAC function?

### HDAC activity is required for IFN-γ induced gene expression

Prior to our study, the use of HDAC inhibitors had firmly established deacetylation as a requirement in antiviral gene expression induced by IFNs [51]. Type I IFNs require at least one class I deacetylase, HDAC1, and it is possible that another HDAC is also involved [22,24]. The type II IFN pathway also requires deacetylation. The HDAC inhibitors trichostatin A, sodium butyrate and suberoylanilide hydroxamic acid prevent IFN-γ inducible Janus kinase 1 activation, STAT1 phosphorylation, nuclear translocation and the activation of a STAT1-responsive luciferase reporter gene [23]. RNAi mediated knockdown and overexpression of HDAC1, HDAC2 and HDAC3 modulates transcription of this same reporter construct. The HDAC inhibitors also lower IRF1 expression induced by IFN-γ.

We found evidence that HDAC3 is recruited to *IRF1 *in response to IFN-γ and that when this fails to occur, the associated histones become hyperacetylated. Our interpretation of these data is that the inappropriate 'openness' of the chromatin template contributes to the loss of definition at the TSS, and to the inefficient processing of *IRF1 *pre-mRNA to the mature mRNA. IRF1 protein is, therefore, not expressed to sufficient levels, and the antiviral state is diminished. So, while HDAC inhibition revealed that acetylation and/or deacetylation events affect many steps in the type II IFN pathway [23], we propose that at least one of these processes involves HDAC3 recruitment to establish and maintain a conducive chromatin environment during IFN-γ induced gene expression.

### Alternative TSSs at the *IRF1 *promoter

CAGE has shown that mammalian TSSs are not only defined by a single well-defined base pair [39]. Rather, mammalian promoters can also initiate transcription from multiple sites spread across a region. The assortment of TSSs in the human genome characterized by CAGE was classified according to shape. The four shape classes are: single dominant peak; broad; bi- or multi-modal; and broad with a dominant peak.

The *IRF1 *gene does not have a typical TATA box, although there is an AT rich sequence starting at -38 relative to the TSS, and it does appear to have a CCAAT box at -99 and a GC box at -162. Its TSS is defined by a single dominant peak that maps to the nucleotide at 131,854,364 on the reverse strand of chromosome 5. This is the same base that defines the TSS in our study and the RefSeq mRNA. The other minor CTSSs (14) map to base +4 and -66 bases from this site. While most of the minor CTSSs are from singleton CAGE tags, the CTSS at -11 and -20 were found in different RNA libraries and, in fact, are the major CTSSs in a RNA library produced from a large intestine malignancy. The alternative TSSs described here are proximal to these TSSs; thus, it is interesting to consider the possibility that loss of TSS fidelity away from the single peak might be generally associated with malignancy, and that epigenetic dysregulation, involving H3K4me2 at promoters, could contribute to this process.

The fact that we could detect aberrant TSS transcripts using a qRT-PCR strategy (Additional File 5) for *GAPDH *is also supported by the CAGE analysis; like *IRF1, GAPDH *has several CTSSs that map upstream of its canonical TSS [39]. Importantly, however, these aberrant TSS *GAPDH *transcripts were not enriched in the MEN1-depleted cells.

## Conclusions

Based on the results of this study, we hypothesize a novel role for MEN1 in which it, as part of the Set1-like MLL1/MLL2 complex, regulates the dimethylation of H3K4. H3K4me2 is then recognized for the proper recruitment of HDAC3, which in turn prevents the hyperacetylation of the chromatin template at *IRF1*, when its transcription is triggered by IFN-γ. Without HDAC3's opposing activity, the chromatin template becomes too 'open' and the information it provides for the regulation of gene expression is compromised, resulting in a poorly defined TSS and inefficient RNA processing. Thus, the post-translational modification of histones modulates not only the transcriptional processes of initiation, elongation and termination, but co-transcriptional processes as well.

## Methods

### Antibodies

H3K4me3 (Abcam ab8580), RNA Pol II Ser2 (Abcam ab5095), LSD1/KDM1 (Abcam ab17721), HDAC3 (Abcam ab7030-50), NCoR (Abcam ab24552) and GAPDH (Abcam ab9485) were from Abcam, Cambridge MA USA. H3K4me2 (Millipore 07-030), Pan H3 CT (Millipore 07-690), Acetyl Histone H3 (Millipore 06-599), HDAC1 (Millipore 17-608) and SMRT (Millipore 17-10057) were from Millipore, Billerica MA USA. RNA Pol II (Santa Cruz sc-899), STAT1 (Santa Cruz sc-345X) and IRF1 (Santa Cruz sc-497) were from Santa Cruz Biotechnology, Santa Cruz CA USA. Menin (Bethyl A300-105A) was from Bethyl Laboratories, Montgomery TX USA. IgG and anti-rabbit or anti-mouse HRP were from Jackson Immunoresearch, West Grove PA USA. Splicing factor 3a antibodies were a gift from Angela Krämer (University of Geneva).

### Cell lines and chemical inhibitors

2fTGH cells were cultured in HyClone DMEM/high glucose medium (HyClone, Rockford IL USA) supplemented with 10% cosmic calf serum and 10% antibiotic/antimycotic (Fisher Scientific, Pittsburgh PA USA). IFN-γ treatment in all cases involved adding IFN-γ (R&D Systems, Minneapolis MN USA 5 ng/mL) to the medium for 30 minutes, replacing with fresh medium and harvesting cells at the indicated times. Cells were treated with 100 μM 2-PCPA for 24 hours (Sigma-Aldrich, St. Louis MO USA) prior to experimentation.

### Transfection of shRNAmir and expression vectors

An inducible pTRIPZ shRNAmir vector targeting menin (*MEN1*) mRNA (RHS4696-99702478), as well as a nonsilencing shRNA vector (RHS4743) were purchased from Open Biosystems, Huntsville AL USA. A second pGIPZ shRNAmir vector targeting menin (RHS4430-98819991, Open Biosystems) also was characterized. Transfection of 2fTGH cells with each was carried out using Arrest-In reagent according to the manufacturer's protocol (Open Biosystems). Puromycin (3 μg/mL) was used to select for stable cell lines. Cells were treated with 2 μg/mL of doxycycline (Fisher Scientific, Pittsburgh PA USA) 24 hours prior to induction to activate the pTRIPZ vector and characterized based on menin protein expression by western blotting. C-terminally FLAG-tagged MEN1 was PCR cloned using the AscI and MluI sites of pCMV6-entry (OriGene PS100001, Origene, Rockville MD USA) and the *MEN1 *cDNA as template (Open Biosystems, IHS1380-97652628).

### ChIP

Crosslinked ChIP was performed as described in [52]. Briefly, 1 × 10^7 ^cells were fixed in 1% formaldehyde for 10 minutes followed by the addition of 0.125 M glycine. Cells were lysed using a douncer and the fixed chromatin was sheared by sonication. For micrococcal nuclease ChIP, 1 × 10^7 ^cells were collected and lysed using a dounce homogenizer. Cells were digested with 100 units/mL of micrococcal nuclease (Worthington, Lakewood NJ USA). Sheared or digested chromatin was then incubated overnight with various antibodies. Pan H3 and IgG were included in all ChIPs as positive and negative controls. Immunoprecipitation was carried out with protein A or G agarose and salmon sperm DNA beads (Millipore). After washing, the chromatin was eluted from the beads and the crosslinks were reversed by heating at 65°C overnight. DNA was treated with RNase A and Proteinase K (5 Prime, Gaithersburg MD USA), purified via phenol and chloroform extraction, precipitated with ethanol overnight and resuspended in tris-ethylenediaminetetraacetic acid buffer. Samples were analyzed by qRT-PCR (Applied Biosystems Carlsbad CA USA) using gene specific primers designed to run the length of the *IRF1 *gene. Primer sequences can be provided upon request. PCR efficiency was determined for all primer pairs before their use. Data are expressed as percentage of input and all experiments were performed in duplicate, if not triplicate. One replicate is shown in Figures 2, 3 and 7. To ensure the statistical significance of differences reported in the ChIP assays, standard errors were calculated for the multiplicates and, if necessary, a Student's t-test confirmed significance, *P *≤ 0.05 [53].

### qRT-PCR

Total RNA was collected using Isol-RNA lysis reagent (5 Prime). RNA was DNaseI (Life Technologies, Grand Island NY USA) treated and extracted with phenol and chloroform. RNA (2 μg) was converted to cDNA using the High Capacity RNA-to-cDNA kit (Applied Biosystems). cDNA was then subjected to Q-PCR (SYBR Green, 7500 FAST Real Time PCR System, Applied Biosystems) using gene specific primers to the intronic or exonic regions of the *IRF1 *gene. In all cases, an RT negative control confirmed no genomic DNA contamination. Primer sequences can be provided upon request. PCR efficiency was determined for all primer pairs before their use by using genomic DNA as template. To ensure the statistical significance of differences reported in the qRT-PCR assays, standard errors were calculated for the multiplicates and a Student's t-test confirmed significance, *P *≤ 0.05 [53]. To quantify aberrant TSS transcripts (n = 2), primers that flank the canonical RefSeq TSS for the *IRF1 *and *GAPDH *(Pol II genes) genes, and the *RN7SK *(Pol III gene) gene were used, as were primers that quantify total mRNA or snRNA transcripts for each of these genes. *IRF1 *aberrant TSS (forward; 5'-CCGCTAAGTGTTTGGATTGC, reverse; 5'-CTCGGGCGCACGTCTT), *IRF1 *total (forward: 5'-AAAGGAGCCAGATCCCAAGAC; reverse: 5'- GGTGGAAGCATCCGGTACAC), *GAPDH *aberrant TSS (forward: 5'- GCGCCCCCGGTTTCTATA; reverse: 5'-GATGCGGCTGACTGTCGAA), *GAPDH *total (forward: 5'-GACAACTTTGGTATCGTGG; reverse; 5'-GGTGGCAGTGATGGCATGG), *RN7SK *aberrant TSS (forward: 5'-TGTAAAGTTGAGACTTCCTTCAGGTT; reverse: 5'- AACCCTGGCGATCAATGG), *RN7SK *total (forward: 5'-TCTTCGGTCAAGGGTATACGAGTAG; reverse: 5'- TACAAATGGACCTTGAGAGCTTGT). Q-PCR primers that could quantify aberrant TSS transcripts for the 45S rRNA that is processed to 18S rRNA (*RN18S1*; a Pol I gene) could not be designed using Primer Express (Applied Biosystems) and so standard RT-PCR was performed, except that a second 25 cycle amplification was performed using a 10% aliquot of the first PCR reaction as template. This primer pair amplifies a product of the correct size when genomic DNA is the template. *RN18S1 *aberrant TSS (forward: 5'- CGGGTTATTGCTGACACGC; reverse: 5'- CCGCGCGCATCCGGAGGCCCAAC), *RN18S1 *total (forward: 5'- GTGCCAAGCAGCCGCGGTAA; reverse: 5'-GGGCATCACAGACCTG).

### RT-PCR

RT was as described above. PCR was performed (20 cycles) using primers designed to *IRF1 *exon 4 (forward: 5'-CTGCCAGATATCGAGGAGGTGAAA-3') and exon 5 (reverse: 5'-TCTTGGCCTTGCTCTTAGCATCTC-3').

### Western blotting

Cells were collected after various treatments and whole cell extract, prepared as described in [54], was subjected to SDS-PAGE (30 μg) and transferred to a nitrocellulose membrane. Immunodetection was performed using the indicated antibodies. A horseradish peroxidase anti-species secondary antibody (1:10,000) was then applied and immunoreactive proteins were visualized using chemiluminescence reagent (Fisher Scientific, Pittsburgh PA USA). Histone acid extraction was carried out as described previously [55]. Bands were quantified with ImageJ software.

### Northern blotting

Total RNA was extracted from *shRNAmir-MEN1 *and *shRNAmir-NS *cells using Isol-RNA lysis reagent (5 Prime). Equal amounts of RNA (20 μg) were electrophoretically separated on a 1.5% agarose-formaldehyde gel. The RNA was transferred to a nylon membrane by capillary action and fixed by baking. The northern blots were probed with [^32^P]-labeled *IRF1 *(Open Biosystems IHS1380-97433442) and *GAPDH *(Open Biosystems IHS1380-97434647) cDNAs prepared using the DECAprime II kit (Ambion Life Technologies, Grand Island NY USA). Phosphor screens were exposed to probed blots, and the band intensity was quantified with Storm 840 Imager and ImageQuant TL software (GE Healthcare, Piscataway NJ USA).

### CPE assay

CPE assays (n = 2) were performed as described in [30]. The titered VSV Indiana strain was a generous gift from Gail Wertz (University of Virginia). *shRNAmir-MEN1 *cells were grown in HyClone DMEM/high glucose medium supplemented with 10% tetracycline-screened fetal bovine serum and 10% antibiotic/antimycotic (Fisher). Cells were cultured with or without 2 μg/mL of doxycycline (Fisher Scientific). Approximately 8000 cells per well were plated in 96-well dishes and allowed to grow overnight. Cells were untreated, treated with IFN-γ (R&D Systems, 10 ng/mL) or IFN-α (R&D Systems, 1000 U/mL) for 12 hours and then infected with various dilutions of VSV for approximately 15 hours. The viral medium was removed, cells were fixed with 5% formaldehyde and stained with 0.05% crystal violet. *shRNAmir-NS *cells did not phenocopy 2fTGH cells in this assay, perhaps due to an off-target effect that becomes relevant in this biological response assay. Therefore *shRNAmir-MEN1 *(-) doxycycline cells were used as the control.

### 5' and 3' RACE-PCR

RNA was isolated from 2fTGH cell lines under various conditions. RACE-PCR was performed using the Gene Racer Kit (Invitrogen). PCR products were generated using a 5' primer or 3' primer provided in the kit, as well as *IRF1 *specific primers designed to exon 3 (forward: 5'-CCCAGCTCCGGAACAAACAGGCATCC-3') for 3'RACE, or exon 3 (reverse: 5'-GGATGCCTGTTTGTTCCGGAGCTGGGC-3') for 5' RACE or exon 10 (forward: 5'-CAGTCACAGACAGAACAGTCAGCAGCCC-3') for 3' RACE. Products were gel purified and cloned into the TOPO vector (pCR4-TOPO). TOP10 competent cells were transformed and plasmid DNA was isolated from multiple clones using the Qiagen Spin Miniprep Kit (Qiagen, Valencia CA USA). DNA was then sequenced with M13F and M13R primers provided in the TOPO cloning kit. Sixteen or seventeen clones were sequenced for each of the different conditions. A Fisher's exact test determined significance.

## Abbreviations

2-PCPA: trans-2-phenylcyclopropylamine; bp: base pairs; CAGE: cap analysis of gene expression; ChIP: chromatin immunoprecipitation; CoREST: RE1-silencing transcription factor co-repressor; CPE: cytopathic effect; CTD: C-terminal domain; CTSS: CAGE tag starting site; DMEM: Dulbecco's modified Eagle's medium; HDAC: histone deacetylase; HMT: histone methyltransferase; hnRNA: heterogeneous nuclear mRNA; IFN: interferon; IgG: immunoglobulin G; kb: kilobases; LSD1: lysine specific histone demethylase 1 isoform a; me2: dimethylation; me3: trimethylation; MEN1: multiple endocrine neoplasia type 1; MLL: mixed lineage leukemia; NCoR: nuclear receptor corepressor; NR: nuclear receptor; PCR: polymerase chain reaction; qRT-PCR: quantitative real time PCR; RACE: rapid amplification of cDNA ends; Set1: Set domain containing 1A; SMRT: silencing mediator of retinoid and thyroid receptors; SnRNP: small nuclear ribonucleoprotein; STAT: signal transducers and activators of transcription; TSS: transcription start site; VSV: vesicular stomatitis virus.

## Competing interests

The authors declare that they have no competing interests.

## Authors' contributions

MAH conceived of and directed the study and wrote the manuscript. LBA designed the experimental strategy, performed the experiments and contributed to the manuscript preparation. SS performed the aberrant TSS RT-PCR assays and HDAC3 complex component ChIP experiments. LML designed and performed the rescue assays. All authors read and approved the final manuscript.

## Supplementary Material

Additional File 1 ***IRF1 *hnRNA transcripts are enriched in the MEN1-depleted cell line. (a-e) **qRT-PCR to quantitate *IRF1 *total and pre-mRNA expression in shRNA-MEN1 and shRNA-NS cell lines that were uninduced (un) or treated with IFN-γ. *IRF1 *expression was normalized to *β-actin *and presented as fold change relative to the uninduced, shRNA-NS condition. Numbers are the base pair location of the primers used on the *IRF1 *gene; see Figure 1c. Error bars are standard error (n = 4). **P ≤ 0.01, *P ≤ 0.05. Click here for file

Additional File 2 **Increased rate of transcription does not account for elevated hnRNA pool in the *shRNAmir-MEN1 *cell line. (a-b) **qRT-PCR to quantitate *IRF1 *pre-mRNA and total RNA expression in shRNA-MEN1 and shRNA-NS cell lines that were uninduced or treated with IFN-γ and collected at indicated time points. *IRF1 *expression was normalized to *β-actin *and presented as fold change relative to the uninduced, non-silencing shRNAmir condition. Error bars are standard error (n = 4). **P ≤ 0.01. Click here for file

Additional File 3 **CPE assay MEN1 protein levels. (a) **MEN1 western blot of whole cell extracts prepared from shRNA-MEN1 cells treated with doxycycline for 24 hours or not. GAPDH served as the loading control. Click here for file

Additional File 4 **Phosphoserine 2 Polymerase II levels are not altered in the MEN1-depleted cell line. (a, b) **Chromatin immunoprecipitation of shRNA-MEN1 and shRNA-NS cell lines treated with IFN-γ for 90 minutes or uninduced using the antibody specific for the phosphoserine 2 form of Pol II. Click here for file

Additional File 5 **Aberrant TSS transcripts at Pol I, Pol II and Pol II genes. (a) **qRT-PCR to quantitate transcripts initiated from a TSS upstream of the canonical TSS for *IRF1, GAPDH *and *RN7SK *(a Pol III gene) in shRNA-MEN1 and shRNA-NS cell lines. *IRF1, GAPDH *and *RN7SK *mRNA transcripts and aberrant TSS transcripts were normalized to *β-actin*; the aberrant TSS transcripts were calculated as a fraction of total mRNA, and presented as fold enrichment relative to the non-silencing shRNAmir condition. Error bars are standard error (n = 2). A student's one-tailed t-test determined significance. *P ≤ 0.05. **(b) **Effective qPCR primers could not be designed at the TSS of the 45S rRNA that is processed to 18S rRNA (*RN18S1*, a Pol I gene) and so endpoint PCR was used to attempt to detect aberrant TSS transcripts. 25 cycles of PCR did not generate an amplicon, nor did a second round of 25 cycles using an aliquot (10%) of the first reaction as template (arrow), but when genomic DNA was used a template an amplicon of the correct size was produced (positive control). This experiment was done for two biological replicate cDNA preparations, though only one is shown. 18S ORF primers detect the *RN18S1 *rRNA. RT+ indicates reverse transcriptase was included in the cDNA reaction while RT- indicates it was omitted. Click here for file
